# Alcoholic Steatosis in Different Strains of Rat: A Comparative Study

**DOI:** 10.4303/jdar/235912

**Published:** 2015-05-25

**Authors:** Kamlesh K. Bhopale, Shakuntala Kondraganti, Harshica Fernando, Paul J. Boor, Bhupendra S. Kaphalia, G. A. Shakeel Ansari

**Affiliations:** Department of Pathology, The University of Texas Medical Branch, Galveston, TX 77555, USA

**Keywords:** alcohol, ethanol, lipids, genes, transcription factors, fatty liver, alcoholic liver disease, rat strains, steatosis

## Abstract

**Background:**

Different strains of rats have been used to study alcoholic liver disease (ALD) while the reason for selecting a particular rat strain was not apparent.

**Purpose:**

The aim of our study was to compare outbred (Wistar) and inbred (Fischer) strains to evaluate pathological, biochemical changes, and gene expression differences associated with ethanol-induced early hepatic steatosis.

**Study Design:**

Male Wistar and Fischer-344 rats were pair-fed for 6 weeks with or without 5% ethanol in Lieber-DeCarli liquid diet. Livers were analyzed for histological and lipid-related differences.

**Results:**

Hepatic midzonal steatosis was mainly found in Wistar rats while Fischer rats showed mostly pericentral steatosis. Increased hepatic steatosis in ethanol-fed Wistar rats is supported by increases in lipids with related genes and transcription factors involved in fatty acid and triglyceride synthesis.

**Conclusion:**

Our data showed that Fischer rats are relatively less prone to ethanol-mediated steatosis with pericentral lipid deposition pattern in the liver which is similar to humans and show no trace level of lipid accumulation in pair-fed controls as observed in Wistar (outbred) strain. Therefore, Fischer rats are better suited for lipid studies in an early development of ALD.

## 1. Introduction

Hepatic steatosis (fatty liver) is a pathological condition that results from excessive accumulation of lipids in the liver. Fatty liver is usually considered benign and reversible, but can progress to steatohepatitis with fibrosis and cirrhosis, or hepatocellular carcinoma with persistent exposure to the etiological agent(s) [1,2,3]. Initially, many alcoholics display no clinical symptoms, but among heavy long-term alcoholic drinkers, approximately 90% develop fatty liver, of which 10%–35% develop irreversible alcoholic hepatitis and about 8%–20% develop cirrhosis [4]. Amongst the liver diseases, the two most commonly diagnosed diseases are alcoholic liver disease (ALD) and nonalcoholic fatty liver disease (NAFLD). NAFLD can be due to high fat diet, nutrient deficiency, and metabolic disorders such as diabetes. Both diseases share a similar fatty liver condition at their early stages. ALD is associated with high morbidity, and nearly 3.8% of all global deaths are reported as a result of excessive alcohol consumption on a regular basis [5]. Alcohol abuse has affected over 10 million people in the USA, and ~ $166 billion a year was spent about a decade ago for treatment [6]. In the present era, the prevalence of ALD is estimated to cost the American economy more than $257 billion [7].

The mechanism(s) and pathway(s) leading to steatosis in ALD are still unclear, primarily due to the lack of a well-defined animal model [8]. However, rats are the commonly used laboratory animals in ALD research, but the choice for a particular rat strain is not specified. The aversion towards alcohol by rats is partially overcome by using a specially formulated Lieber-DeCarli liquid diet where the defined percentage of alcohol can be added [9]. In ALD research, Wistar and Sprague Dawley (outbred strains) rats are most commonly used compared to Fischer-344 (an inbred rat strain). It is important to take into consideration the responsiveness of rat strains to a given chemical or pharmaceutical agent that showed differential sensitivity to target organs including liver [10]. Inbred strain is animal of choice because of homozygosity, and thus the biological effect can be evaluated using fewer animals. For fatty acid metabolism studies in disease processes (e.g., ALD), more than one strain is needed for interpretation of data in humans [11]. The altered lipid profile is one of the key features of fatty liver among alcoholics at early stages. Therefore, the primary aim of our study was to compare alcoholic steatosis in Wistar (outbred strain) and Fischer (inbred strain) rats under similar condition that may help us in selecting a rat strain depending upon objective such as identification of lipids biomarker and therapeutic targets.

## 2. Materials and methods

### 2.1. Animals and diet

Wistar and Fischer-344 rat strains (male; 6–7 weeks old) were purchased from Harlan (Indianapolis, IN, USA). Animal experimentation was performed in accordance to the protocol approved by Institutional Animal Care and Use Committee of The University of Texas Medical Branch, USA, and followed the NIH Guide for care and use of laboratory animals. Rats were housed in humidity- and temperature-controlled animal room with an automatic controlled 12-hour light/dark cycle throughout the experiment. After 1 week of acclimatization, rats of both strains were randomly divided into their two respective groups: group 1 was pair-fed on Lieber-DeCarli liquid diet as a control (Dyets Cat. #710260, Dyets, Inc.; Bethlehem, PA, USA), and group 2 was fed on Lieber-DeCarli liquid diet containing 5 g/dL ethanol for 6 weeks. Each strain had groups consisting of a minimum of 6 rats. Diets were freshly prepared daily according to the manufacturer’s instructions, and caloric values derived from ethanol was substituted with maltose-dextrin (Dyets Cat. #402851) for pair-fed controls [12,13,14]. Since rats have aversiveness for ethanol, the amount of ethanol fed to the rats in the diet was ramped from 1 g/dL to 5 g/dL in a one-week period, and then maintained at 5 g/dL till the end of experiment for 6 weeks. The control or ethanol diet is nutritionally complete with total calories as carbohydrate 11% + ethanol or maltose-dextrin 36%, protein 18%, and fat 35%. Freshly prepared isocaloric control or ethanol diet was given daily to animals, and diet intake of each rat was recorded daily. Rats were monitored for their general health, checked for any signs of distress or morbidity daily, and weighed once a week. Animals were euthanized at the end of the 6th week by intraperitoneal injection of pentobarbital sodium (Nembutal, 100 mg/kg body weight). Blood was withdrawn from the heart in heparinized tubes, centrifuged at 1000 g for 10 min, and the plasma obtained was stored at −80 °C until further analysis.

### 2.2. Liver pathology

The liver from each rat was harvested, grossly examined, and weighed. Three small pieces from the left liver lobe were cut (one immediately frozen in liquid nitrogen for lipid analysis, a second stored in RNAlater, and a third fixed in 10% buffered formalin for Hematoxylin and Eosin (H&E) staining) and the remainder of the liver was stored at −80 °C. Steatosis was graded by histological examination of Oil Red ‘O’ stained liver sections specific for lipid deposition in the hepatocytes, according to the Tsukamoto grading scale 1–4 based on percent of hepatocytes with fat (0%–25% of cells involved as grade 1; 25%–50%, grade 2; 50%–75%, grade 3; > 75%, grade 4) [12,15].

### 2.3. Liver injury markers

The plasma samples were analyzed for hepatic injury markers using commercial kits of alanine aminotransferase (ALT/SGPT Liqui-UV kit), alkaline phosphatase (ALP Liquicolor kit), and lactic dehydrogenase (LDH Liqui-UV kit) from Stainbio Laboratory, Boerne, TX, USA, and aspartate aminotransferase (AST/SGOT Reagent kit) from TECO Diagnostics, Anaheim, CA, USA.

### 2.4. Biochemical analysis of hepatic lipids

Lipids were extracted from the livers with methyl-*tert*-butyl ether (MTBE), as described previously [13,14,16]. Briefly, 250 mg of liver sample was homogenized in methanol (1.5 mL), and MTBE (5.0 mL) was added and shaken for 1 h at room temperature. Subsequently, high purity water was added (1.25 mL), mixed, and allowed to stand for 10 min, centrifuged at 1,000 g for 10 min, and the upper organic layer was collected. The aqueous layer was re-extracted with MTBE/methanol/water mixture (10/3/2.5v/v/v; 2 mL) and the combined organic layers were dried under nitrogen. The extracted dried lipids were weighed, dissolved in a mixture of triton X-114/methanol (2:1v/v, 60 *μ*L), and stored at −20 °C until analyzed. The lipids were analyzed using commercially available kits (Wako Diagnostics, Wako Chemicals USA, Inc.; Richmond, VA, USA) for triglyceride (L-Type TGH), total cholesterol (Cholesterol E), free cholesterol (Free Cholesterol E), and free fatty acids (nonesterified fatty acid, NEFA; HR series NEFA-HR (2)).

### 2.5. RNA extraction

RNA was extracted from liver tissues and stored in RNAlater using RNAqueous kit, following the manufacturer’s protocol (Ambion, Austin, TX, USA). Briefly, 25 mg of liver tissue was homogenized in tissue lysis buffer (600 *μ*L) and equal volume of 64% ethanol was added, mixed, applied to a filter cartridge and centrifuged at 12,000 g for 1 min at room temperature, washed with buffer, and recentrifuged. The filter cartridges were transferred to a fresh tube, and RNA was eluted with 50 *μ*L of preheated (70 °C) elution buffer. RNA concentration was determined by NanoDrop ND-1000 UV-Vis Spectrophotometer (Nanodrop Technologies, Inc., Wilmington, DE, USA) and the RNA integrity was analyzed by Bioanalyzer System (Agilent Technologies, Santa Clara, CA, USA).

### 2.6. cDNA and real-time qPCR (quantitative polymerase chain reaction)

Reverse transcription of extracted RNA to cDNA was carried out by TaqMan Reverse Transcription Reagent (Applied BioSystems, Carlsbad, CA, USA). The reaction mixture was incubated for 10 min at 25 °C, followed by reverse transcription for 30 min at 48 °C and inactivation of reverse transcriptase for 5 min at 95 °C in a thermo cycler (Bio-Rad Tertrad2, Hercules, CA, USA). Quantitative PCR was done with 10 *μ*L PCR reactions in a 364-well plate using SYBR Green I Master mix (Roche Applied Science, Indianapolis, IN, USA) and primers from Integrated DNA Technologies (IDT, Coralville, IA, USA) on a Roche LightCycler 480 (Roche). The PCR reaction mixture was preincubated at 95 °C for 5 min to activate DNA polymerase, followed by 45 cycles of amplification at 95 °C for 10 s, 60 °C for 10 s, and 72 °C for 15 s. At the end of the final cycle, a melting curve analysis was performed for each sample.

### 2.7. Statistical analysis

The data were analyzed as mean ± SD and subjected to Student’s *t*-test or Student-Newman-Keuls multiple comparisons. Differences between groups were considered significant at *P* ≤ .05. For gene analysis, the relative expression of the genes was calculated by comparative *C*_T_ method and expressed as fold change with L19 as endogenous control, where fold change = 2^−ΔΔ*C*_T_^ and ΔΔ*C*T = (*C*_T ethanol_ − *C*_TL19_) − (*C*_T control_ − *C*_TL19_) [17]. The data were normalized with L19 as an internal control and analyzed by comparing to fold changes in the samples obtained from alcohol-treated versus respective control rats. However, for 3-Hydroxy-3-methylglutaryl-CoA (HMG-CoA) reductase and sterol-regulatory element-binding protein 2 (SREBP-2), data were normalized with 18S endogenous control and the fold change in expression between the ethanol and control rats is calculated by ethanol/control using equation 2^−ΔΔ*C*_T_^.

## 3. Results

### 3.1. Body weights and liver weights

The ethanol-fed groups gained relatively less body weight than their respective controls, but the differences were not statistically significant (data not shown). Liver weight/body weight ratio significantly increased by ~ 12% in ethanol-fed Wistar rats as compared to the controls, whereas only marginally increased in Fischer rats and was not statistically significant.

### 3.2. Liver histopathology

**Figure 1:**
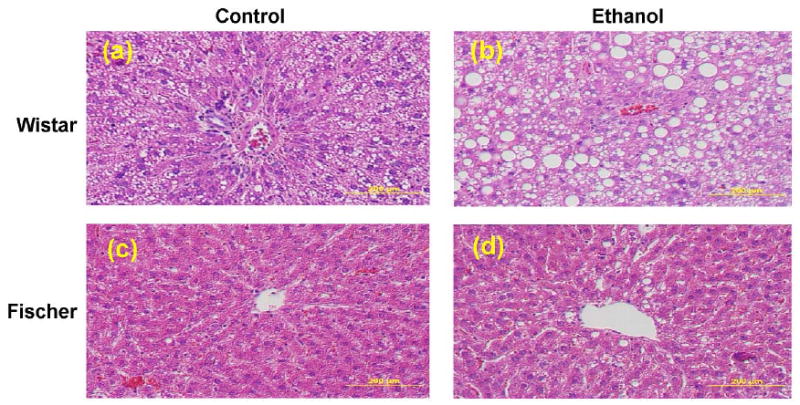
Ethanol-induced hepatic steatosis in rats. Histological sections of livers were stained with Hematoxylin-eosin (H&E). Photomicrographs of representative liver sections of rats shown; Wistar (a), (b), and Fischer (c), (d); control diet feeding produced mild steatosis only in Wistar rats (a). Ethanol feeding for 6 weeks resulted in significant steatosis without inflammation in all rats [Wistar (b), Fischer (d)] with maximum micro- and macrovacuolization in Wistar (b). All photomicrographs are at the original magnification ×200.

**Figure 2:**
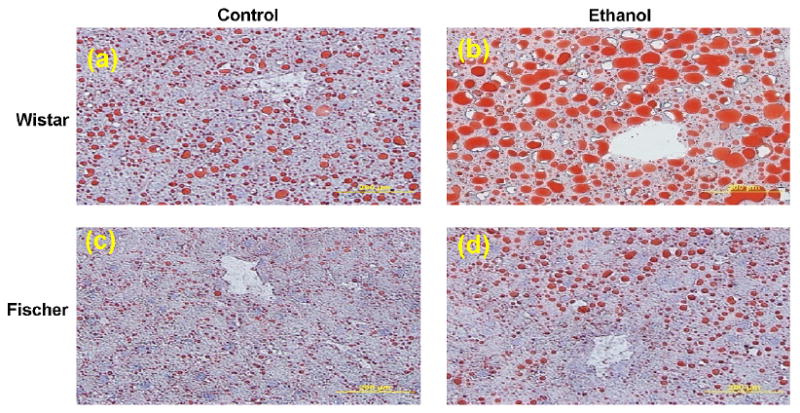
Increased fat deposition was observed in Oil Red ‘O’ stained liver sections of ethanol-fed rats for 6 weeks; Wistar (upper panel), control (a) and ethanol (b), Fischer strain (lower panel) fed control (c) and ethanol diet (d). Wistar rats exhibit more fatty changes (b) than Fischer (d) when fed ethanol diet. Control diet caused measurable fatty changes only in Wistar rats (a). All photomicrographs are at the original magnification ×400.

**Figure 3:**
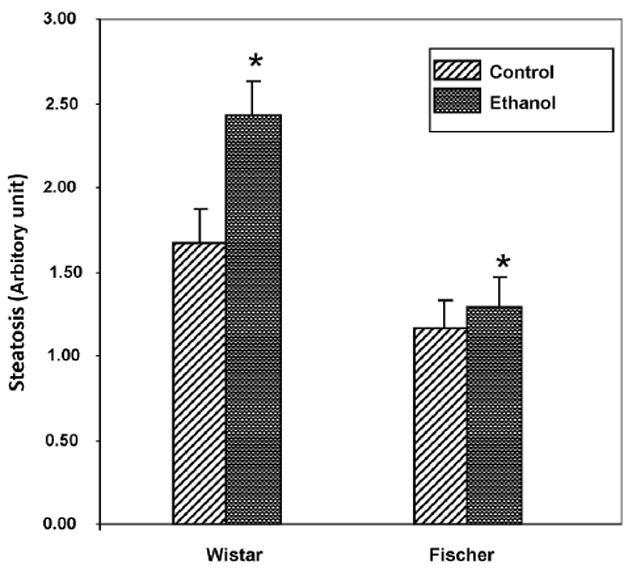
Wistar rats showed higher steatosis score compared to Fischer strain. Steatosis score was assigned to liver sections of rats using Tsukamoto histopathological grading 1–4 scale [15]. Ethanol-fed diet group is compared with control-fed diet group. Three randomized fields of liver sections of each animal examined under ×100 magnification, and percent of hepatocytes with fat deposition is graded as 1%–25% Grade 1, 25%–50% Grade 2, 50%–75% Grade 3, and > 75% Grade 4. Values represent mean±SD (*n* = 6 or 7 animals/group in each strain). Asterisk indicates *P* values ≤ .05 as significant difference between ethanol and respective controls.

Steatosis (macro- and microvesicular fatty changes) of hepatocytes was predominantly seen in the ethanol-fed Wistar rats (Figure 1). The controls group of Wistar rats also showed mild steatosis in the liver sections. Hepatic steatosis in ethanol-fed Fischer was milder than in Wistar rats. However, the steatosis pattern was dissimilar between strains in the present study, as observed in Oil Red ‘O’ stained liver sections (Figure 2). Wistar rats had midzonal pronounced fatty infiltration ranging from micro- to macrovacuolization; whereas Fischer rats showed pericentral fatty changes. Liver histology of ethanol-fed rats did not show inflammation (Figure 1). Fatty change (cytoplasmic vacuolization) observed in the liver sections was scored on a 1–4 grading scale. Wistar rats showed significantly greater steatosis than Fischer rats fed ethanol diet (Figure 3). This histopathological grading data paralleled the quantity of extracted lipids (Supplementary Figure 1).

### 3.3. Liver injury markers and lipid biochemical analysis

Plasma ALT, AST, ALP, and LDH levels increased in both ethanol-fed groups (Supplementary Table 1).

Biochemical analysis indicated variations in hepatic lipid levels in both strains of ethanol-fed rats as compared to their respective pair-fed controls (Table 1). Wistar rats showed maximum accumulation of lipids in their livers as compared to Fischer rats. Triglyceride accumulation in the liver was increased by 1.8 fold and 1.38 fold in Wistar and Fischer rats, respectively. Hepatic total cholesterol level was more increased in Fischer (1.56 fold) as compared to Wistar strain (1.27 fold). Free cholesterol was also increased in both ethanol-fed strains. Free fatty acids (nonesterified fatty acid, NEFA) were highly increased in Wistar, but not in Fischer strain indicating more availability of free fatty acids for triglyceride synthesis.

### 3.4. Genes and transcription factors

The transcriptional regulations of selected genes involved in lipid synthesis, ethanol metabolism, and signal transduction were analyzed by qPCR, and differences observed in gene expression in ethanol-diet-fed Wistar and Fischer rats are described in the following sections.

#### 3.4.1. Genes involved in alcohol metabolism

Alcohol dehydrogenase (ADH) 1 and cytochrome P450 (CYP) 2E1 are the major genes responsible for alcohol metabolism; both of them were upregulated in Wistar rats, whereas ADH1 was upregulated in Fischer rats with no change in CYP2E1 expression (Table 2).

#### 3.4.2. Genes involved in fatty acid, triglyceride, phospholipid, and cholesterol biosynthesis

Genes involved in fatty acid biosynthesis analyzed in the present study were upregulated in Wistar rats indicating an increased fatty acid synthesis while downregulated in Fischer rats (Table 3). Diacylglycerol *O*-acyltransferase 1 (DGAT-1) gene involved in the triglyceride biosynthesis showed a differential pattern with increased expression in Wistar, and little change in Fischer rats. Both Betaine-homocysteine *S*-methyltransferase (BHMT) and phosphatidylethanolamine *N*-methyltransferase (PEMT) were decreased in Wistar rats and increased in Fischer rats (Table 4). Cholesterol-associated gene 3-hydroxy 3-methylglutaryl CoA (HMG-CoA) reductase increased more in Fischer than in Wistar rats (Table 4).

#### 3.4.3. Genes involved in fatty acid oxidation and inflammation

Both carnitine palmitoyltransferase (CPT)-1*α* and acetyl-CoA carboxylase (ACC) *β* were decreased in both strains of rats, indicating impaired oxidation and acetyl-CoA formation, needed for fatty acid biosynthesis (Table 5). The transcription factor, sterol regulatory element-binding protein 1 (SREBP-1), which regulates genes involved in lipid synthesis, was decreased in both strains, probably due to increased activation of AMP-activated protein kinase (AMPK) (Table 6). Peroxisome proliferator-activated receptors (PPAR)-*α* and (PPAR)-*γ* were expressed differentially. PPAR-*α* was decreased more in Wistar as compared to Fischer strain, whereas PPAR-*γ* increased more in Fischer than in Wistar strain (Table 6). Nuclear factor of kappa light polypeptide gene enhancer in B-cells1 (NF-kB), a transcription factor involved in the regulation of inflammation, was not distinctly elevated either in Wistar or in Fischer ethanol-fed rats (Table 6).

## 4. Discussion

Alcoholic fatty liver is associated with altered lipid metabolism and lipid homeostasis [18]. Lipid accumulation in hepatocytes was evident in ethanol-fed groups as reported before [13,14,19,20]. However, the pattern of fatty changes in the liver differed with the rat strain: midzonal in Wistar whereas pericentral in Fischer, similar to that reported in humans [21]. A small lipid accumulation observed in the liver of control Wistar rats could be attributed to their propensity of lipid accumulation from fat content of the control diet. Elevated liver enzymes in plasma were consistent with ethanol-induced mild hepatic injury in rats.

Triglyceride accumulation in the liver could be attributed to increased biosynthesis of fatty acid and decreased fatty acid oxidation in the liver and/or increased mobilization of fatty acids from adipose tissue due to ethanol exposure [22,23,24,25]. Increased cholesterol content in the hepatocytes could be due to de novo synthesis [26]. It is also possible that ethanol-induced endoplasmic reticulum stress could elevate intracellular cholesterol biosynthesis [27].

Chronic ethanol consumption is known to increase hepatic fatty acids and triglycerides in humans and rodents [25,28,29,30,31,32]. Ethanol is metabolized in the liver by two major gene products, ADH1 and CYP2E1. The differences observed in the expression of these two genes in the liver of Wistar and Fischer rats could be contributing via oxidative pathway of ethanol metabolism [33].

Ethanol stimulates hepatic fatty acid synthesis [25]. In the present study, we observed an increased expression of fatty acid synthesis genes mainly in ethanol-fed Wistar strain. Similarly, increased expression of hepatic fatty acid synthase (FAS) in ethanol-fed Wistar rats is consistent with increased expression of stearoyl-coenzyme A desaturase1 (SCD1), which favors the formation of monounsaturated fatty acids for storage [34]. Similarly, both SCD1 and SCD2 were distinctly elevated in ethanol-fed Wistar rats which could likely be contributing to higher steatosis, whereas corresponding decreased expression of gene in Fischer strain could be attributed to lower steatosis as observed in this study. Transcription factor SREBP-1 is involved in the activation of genes associated with fatty acid metabolism de novo lipogenesis and cholesterol biosynthesis [35]. However, SREBP-1 was decreased in ethanol-fed rats in our study which may be via the activation of AMP-dependent protein kinase (AMPK) phosphorylation which inhibits SREBP-1 [32,36,37,38]. AMPK plays a key role in regulating the effects of ethanol on hepatic SREBP-1 activation, fatty acid metabolism, and the development of alcoholic fatty liver [32]. Increased expression of AMPK and decreased expression of SREBP-1 as observed are in agreement with the literature linking AMPK and SREBP-1 [38,39,40,41]. SREBP-2 regulates the genes of cholesterol biosynthesis and metabolism in hepatocytes [26, 42]. In ethanol-fed rats, HMG-CoA reductase gene was highly expressed in Fischer strains suggesting an enhanced de novo cholesterol synthesis in the liver which is associated with cholesterol biosynthesis and activated SREBP-2 in the liver, and reduced bile acid excretion, suggesting that either/or both pathways may contribute to elevated hepatic cholesterol levels [26,43].

PPAR-*α* inhibition by ethanol results in reduction of fatty acid oxidation, and decreased expression of PPAR-*α* contributes to hepatic steatosis in ethanol-fed Wistar rats [44,45,46,47]. Overexpression of transcription factor PPAR-*γ* in the livers of ethanol-fed Fischer rats perhaps contributes to hepatic steatosis [48,49]. Further, hepatic steatosis observed in ethanol-fed rats without inflammation explains lack of NF-kB expression which is consistent with published literature [13,14,19,20].

Increased accumulation of lipids in the liver could be cumulative effects of (i) an increased DGAT-1 expression and (ii) a decreased formation of phosphatidylcholine due to decreased expression of BHMT and PEMT with accumulation of fat in hepatocytes in Wistar rats as reported by an earlier report [50]. This trend slows down and decreases the excretion of triglycerides via very low density lipoprotein [51]. Further, decreased expression of CPT-1*α* and ACC-*β*, involved in *β*-oxidation of fatty acid observed in both strains, indicates decreased *β*-oxidation which could lead to increased triglycerides, and thus favoring of hepatic steatosis [52]. Ethanol-induced hepatic steatosis in Fischer rats appeared to be favored by lipid uptake and adipogenesis probably due to decreased PPAR-*α* and increased PPAR-*γ* expression.

In summary, Wistar is found to be more susceptible to ethanol-induced hepatic steatosis as compared to Fischer rats. Generally, the differences observed among various strains could be due to several factors such as in ethanol consumption, nutritional status, ethanol metabolism, and genetic makeup [2,3,33,53,54,55,56,57]. Ethanol-induced hepatic steatosis in Wistar rats is associated with increased fatty acid and triglyceride synthesis, decreased *β*-oxidation of fatty acids, and decreased formation of phosphatidylcholine favoring the lipid accumulation (steatosis). These results support the differences observed in hepatic steatosis and related gene expression in inbred and outbred strains of rats. Furthermore, it appears that increases or decreases in the gene expressions that are involved in the synthesis or degradation of fatty acids, cholesterol, and/or triglycerides may be due to differential regulation at the post-transcriptional and post-translational levels, as reported earlier [58]. It is important to note that differential gene expression as observed in two different strains of rats at an early stage of ALD could be adaptive responses to protect the liver at an early stage of ethanol-induced hepatic steatosis. This contention is supported by lipidomic studies where Fischer rats provide a clear difference between treated and pair-fed controls at least in early phase of exposure to alcohol (see [13,14] and unpublished studies). It appears from the present study that Fischer rat strain could be a preferred model for lipidomic studies because of moderate progress of ethanol-induced hepatic steatosis, lower propensity to a high fat diet, and histological changes in the liver similar to humans.

## Supplementary Material

Supp Fig.1

Supp.Table 1

## Figures and Tables

**Table 1:** Hepatic lipids measured in the different strains of rats fed 5% ethanol in the Lieber-DeCarli liquid diet for 6 weeks. Values are expressed as mean±SD.

Parameter	Diet	Wistar	Fischer
Triglyceride (mg/g liver)	Control	31.08±4.26	22.97±2.58
	Ethanol	56.12***±3.41	31.79***±2.22

Total cholesterol (mg/g liver)	Control	8.32 ±0.98	8.46±1.18
	Ethanol	10.59*±2.88	13.20***±0.81

Free cholesterol (mg/g liver)	Control	4.52±0.97	1.81±0.17
	Ethanol	6.27**±1.59	3.67**±0.80

Free fatty acid (*μ*moL/g liver)	Control	39.16 ±2.36	39.56 ±7.24
	Ethanol	61.24***±10.68	48.20±3.52

* *P* value ≤ .05;

** ≤ .01,

*** ≤ .001.

*n* = control 6 rats and ethanol-fed 7 rats in each strain.

**Table 2:** Major genes involved in alcohol metabolism (fold changes compared to corresponding pair-fed controls; *n* = 4–5).

Gene	NM number, Primer sequence	Wistar, fold change (2^−Δ*C*_T_^)	Fischer, fold change (2^−Δ*C*_T_^)
Alcohol dehydrogenase 1	NM_019286.3	**1.34**	**1.57**
	F: TGACACCATGACTTCTGCCC	C 1.62±0.28	C 2.41±0.63
	R: CGCTTACACCGCATGCTG	E 2.17±0.88	E 3.78±0.66

Cytochrome p450 2E1	NM_031543.1	**1.26**	**1.00**
	F: CTGCCCCCAGGACCTTTC	C 0.27±0.05	C 1.18±0.18
	R: GCGCTTTGCCAACTTGGT	E 0.34±0.09	E 1.18±0.17

C = control 2^−Δ*C*_T_^ value mean±SD.

E = ethanol 2^−Δ*C*_T_^ value mean±SD.

**Table 3:** Major genes involved in fatty acid synthesis (fold changes compared to corresponding pair-fed controls; *n* = 4–5).

Gene	NM number, Primer sequence	Wistar, fold change (2^−Δ*C*_T_^)	Fischer, fold change (2^−Δ*C*_T_^)
Acetyl-CoA carboxylase alpha	NM_022193.1	**2.30**	**0.86**
	F: TGCTCCGGCAGTACCTGC	C 0.46±0.06	C 1.61±0.48
	R: GTCGTAGTGGCCATTCTGAAA	E 1.06±0.34	E 1.38± 0.04

Malonyl-CoA decarboxylase	NM_053477.1	**1.30**	**0.96**
	F: GAAGCACCGATACGCCCTC	C 0.36±0.03	C 1.35±0.16
	R: CCGTTCTGCAGGTGGAAGTT	E 0.50±0.16	E 1.30±0.20

Fatty acid synthase	NM_017332.1	**1.41**	**0.52**
	F: AGATCCTGGAACGTGAACATGA	C 1.08±0.42	C 2.0±0.86
	R: GCCGTACTTCACGAATGGGT	E 1.52±0.55	E 1.04±0.08

Stearoyl CoA desaturase 1	NM_139192.2	**2.58**	**0.61**
	F: TTCCTCATCATTGCCAACACC	C 0.31±0.09	C 1.29±0.49
	R: CATTCATACACATCGTTCTGGA	E 0.80±0.21	E 0.78±0.19

Stearoyl CoA desaturase 2	NM_031841.1	5.09*	**0.76**
	F: CCCTGAGGCTCTTTCTCATCA	C 0.34±0.07	C 2.94±1.21
	R: ACACATCGTTCTGGAATGCCA	E 1.73±0.38	E 2.23±0.52

C = control 2^−Δ*C*_T_^ value mean±SD.

E = ethanol 2^−Δ*C*_T_^ value mean±SD.

* *P* value ≤ .05.

**Table 4:** Genes involved in triglyceride, phosphatidylcholine, and cholesterol biosynthesis (fold changes compared to corresponding pair-fed controls; *n* = 4–5).

Gene	NM number, Primer sequence	Wistar, **fold change** (2^−Δ*C*_T_^)	Fischer, **fold change** (2^−Δ*C*_T_^)
Diacylglycerol *O*-acyltransferase 1	NM_053437.1	**1.48**	**1.16**
	F: GGTGCCCTGACAGAGCAGAT	C 0.64±0.02	C 1.12±0.16
	R: CAAACAGGGAACCCACTGGA	E 0.95±0.13	E 1.3±0.05

Betaine-homocysteine *S*-methyltransferase	NM_030850.1	**0.70**	**1.25**
	F: AGAATTCCCCTTTGGATTGGA	C 0.61±0.12	C 2.28±0.66
	R: TGAATATCCCATCTGGTGGCA	E 0.43±0.13	E 2.86±0.26

Phosphatidylethanolamine *N*-methyltransferase	NM_013003.1	**0.91**	**1.27**
	F: ACTTATGCACGCCAGCCCTA	C 0.86±0.10	C 1.71±0.30
	R: AGACGAGTGCCACCAGCAC	E 0.78±0.14	E 2.17±0.04

3-Hydroxy-3-methylglutaryl-CoA reductase	NM_013134.2	**0.91**	1.32*
	F: CTACATCCGTCTCCAGTCCAAAAC	C 1.17±0.71≠	C 1.21 ± 0.23≠
	R: TGACCGCCAGAATCTGCAG	E 1.016±0.49≠	E 1.60±0.20≠

C = control 2^−Δ*C*_T_^ value mean±SD.

E = ethanol 2^−Δ*C*_T_^ value mean±SD.

≠ Value obtained using equation 2^−ΔΔ*C*_T_^.

* *P* value ≤ .05.

**Table 5:** Genes involved in the oxidation of fatty acids (fold changes compared to the corresponding pair-fed controls; *n* = 4–5).

Gene	NM number, Primer sequence	Wistar, fold change (2^−Δ*C*_T_^)	Fischer, fold change (2^−Δ*C*_T_^)
Carnitine palmitoyltransferase 1*α*	NM_031559.2	**0.66**	**0.60**
	F: CCACAAATTACGTGAGTGACTG	C 1.30±0.06	C 1.25±0.17
	R: CCCCGCAGGTAGATATATTCTT	E 0.86±0.19	E 0.75±0.16

Acetyl-CoA carboxylase *β*	NM_053922.1	**0.49**	**0.65**
	F: GGTTGTAACGAGGTGGGCAT	C 1.54±0.30	C 1.06±0.14
	R: GGTTGTAACGAGGTGGGCAT	E 0.75±0.26	E 0.69±0.06

C = control 2^−Δ*C*_T_^ value mean±SD.

E = ethanol 2^−Δ*C*_T_^ value mean±SD.

**Table 6:** Transcription factors involved in lipid metabolism (fold changes compared to corresponding pair-fed controls; *n* = 4–5).

Gene	NM number, Primer sequence	Wistar, fold change (2^−Δ*C*_T_^)	Fischer, fold change (2^−Δ*C*_T_^)
Sterol-regulatory element-binding protein 1 (SREBP-1)	XM_001075680.2	**0.57**	**0.87**
	F: GCGGCTGTCGTCTACCATAAG	C 1.25±0.18	C 1.10±0.26
	R: GTACTTGCCCATGGCATGC	E 0.71±0.21	E 0.96±0.317

Sterol-regulatory element-binding protein 2 (SREBP-2)	NM_001033694.1	**1.08**	**1.15**
	F: ACCTACCACGCGTCAGGC	C 1.03±0.25≠	C 1.02±0.20≠
	R: CGCCATTAGTCGAACAGTTGC	E 1.12±0.21≠	E 1.17±0.36≠

AMP-dependent protein kinase	NM_023991.1	**3.38**	**1.02**
	F: CCCCTTGAAGCGAGCAACT	C 0.29±0.05	C 0.98±0.06
	R: TTAAACCATTCATGCTCTCGTATGT	E 0.98±0.27	E 1.00±0.18

Peroxisome proliferator-activated receptor *α*	NM_013196.1	**0.77**	**0.99**
	F: CTAGCAACAATCCGCCTTTTG	C 1.28±0.16	C 1.44±0.24
	R: GCCATGCACAAGGTCTCCAT	E 0.98±0.19	E 1.43±0.14

Peroxisome proliferator-activated receptor *γ*	NM_001145366.1	**1.21**	2.58*
	F: CGGTTTCAGAAGTGCCTTGC	C 0.86±0.11	C 1.20±0.10
	R: CAAACCTGATGGCATTGTGAGA	E 1.04±0.12	E 3.1±0.68

Nuclear factor of kappa light polypeptide gene enhancer in B-cells1	XM_342346.4	**1.07**	**1.37**
	F: TTGCTGCCTCTCTCGTCCTC	C 0.58±0.41	C 1.77±0.32
	R: CTCGGAGCTCATCTATGTGCTGT	E 0.62±0.15	E 2.42±0.29

C = control 2^−Δ*C*_T_^ value mean±SD.

E = ethanol 2^−Δ*C*_T_^ value mean±SD.

* *P* value ≤ .05.

≠ Value obtained using equation 2^−ΔΔ*C*_T_^.
