# Supplementary figures and images for: Alcoholic Steatosis in Different Strains of Rat: A Comparative Study
Source: J Drug Alcohol Res. Author manuscript; Available in PMC 2016 May 20. (PMC4874529; doi:10.4303/jdar/235912)

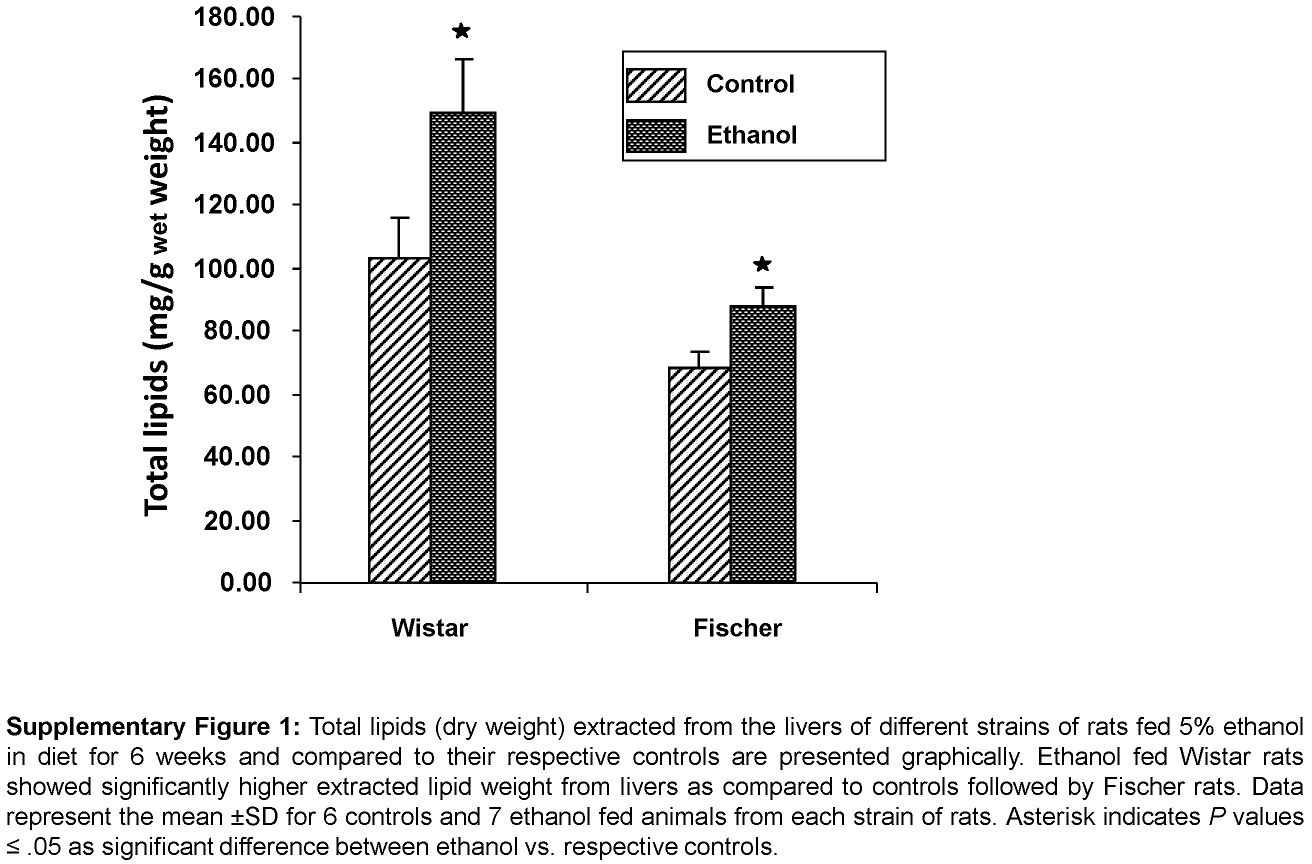

Supplement: Supp Fig.1 [file NIHMS782290-supplement-Supp_Fig_1.tif]

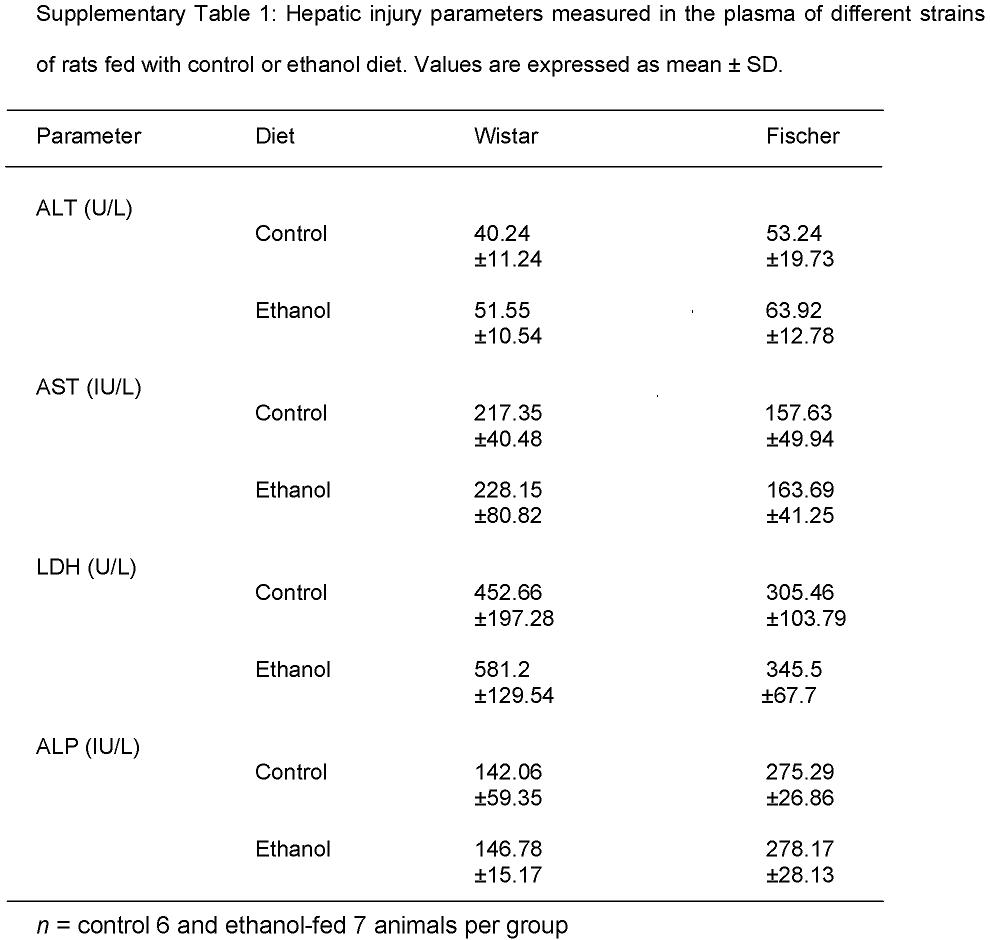

Supplement: Supp.Table 1 [file NIHMS782290-supplement-Supp_Table_1.tif]
